# Draft genome sequences of four potential new species of the genus Bradyrhizobium isolated from root nodules of native legumes in Costa Rican forests

**DOI:** 10.1099/acmi.0.000990.v3

**Published:** 2025-05-16

**Authors:** Rachelle Fernández-Vargas, Sara Jiménez-Alpízar, Valeria Leandro-Arce, Bradd Mendoza-Guido, Keilor Rojas-Jimenez

**Affiliations:** 1 Escuela de Biología, Universidad Costa Rica, San José, Costa Rica; 2 Programa de Posgrado en Microbiología, Parasitología, Química Clínica e Inmunología (PPMPGCI), Universidad Costa Rica, San José, Costa Rica; 3 Programa de Posgrado en Biología (PPB), Universidad de Costa Rica, San José, Costa Rica; 4 Centro Nacional de Innovaciones Biotecnológicas (CENIBiot), CeNAT-CONARE, San José, Costa Rica; 5 Instituto de Investigaciones en Salud, Universidad de Costa Rica, San José, Costa Rica

**Keywords:** bacteria, *Fabaceae*, nitrogen fixation, nodulation, *Rhizobium*, symbiosis

## Abstract

Here, we report the draft genome sequences of four *Bradyrhizobium* spp. isolates obtained from root nodules of the native legumes *Pentaclethra macroloba*, *Chamaecrista nictitans*, *Erythrina fusca* and *Zygia engelsingii* in tropical forests of Costa Rica. Genomes ranged from 8.6 to 9.8 Mb with GC contents between 62.8% and 63.8%. Phylogenomic analysis, along with average nucleotide identity (ANI) and digital DNA–DNA hybridization (dDDH) comparisons, confirmed that these isolates represent potential new species. ANI values ranged from 88.3% to 90.3%, and dDDH values from 28.8% to 41.8%, compared with their closest *Bradyrhizobium* species. Functional annotation revealed some genes related to nitrogen fixation (*nifA*, *nifB*, *nifH*) and nodulation capacity (*nodB*, *nodC*, *nodJ*). These results provide insights into the diversity and symbiotic capabilities of *Bradyrhizobium* in tropical ecosystems.

## Data summary

The raw read sequences of the four *Bradyrhizobium* isolates have been deposited in GenBank, under the BioProject accession no. PRJNA1084876 and Sequence Read Archive (SRA) accession no. SRP493729. The sample accession details are as follows: *Bradyrhizobium* sp. LEGMi-117d (BioSample: SAMN44800723, SRA: SRR31439459, 16S rRNA: PP439200), *Bradyrhizobium* sp. LEGMi-161a (BioSample: SAMN44800724, SRA: SRR31439458, 16S rRNA: PP439223), *Bradyrhizobium* sp. LEGMi-163b (BioSample: SAMN44800725, SRA: SRR31439457, 16S rRNA: PP439225) and *Bradyrhizobium* sp. LEGMi-207a (BioSample: SAMN44800726, SRA: SRR31439456, 16S rRNA: PP439252).

## Announcement

Legumes have established symbiotic relationships with nitrogen-fixing bacteria, enabling them to thrive in nutrient-poor soils and contributing significantly to ecosystem sustainability [1,2]. Among these bacteria, *Bradyrhizobium* (*Nitrobacteraceae*, *Alphaproteobacteria*) is a key genus involved in symbiotic nitrogen fixation, exhibiting notable diversity, particularly in tropical environments [3,4]. However, knowledge about its genomic diversity, particularly in tropical regions and less-studied legumes, remains limited. To help address this gap, we analysed *Bradyrhizobium* isolates obtained from nodules of native leguminous plants in Costa Rica, a region with rich but underexplored microbial diversity.

In this work, we characterized four *Bradyrhizobium* isolates obtained from *Fabaceae* plants in Costa Rica. The four native *Fabaceae* species from tropical Costa Rican forests are as follows: *Pentaclethra macroloba*, *Chamaecrista nictitans*, *Erythrina fusca* and * Zygia engelsingii*. The isolation and DNA extraction processes were performed following the protocol described by Rojas-Jimenez *et al*. [5]. Briefly, root nodules were collected from young trees (30–100 cm in height, 1–3 years old) from three nurseries across different regions of Costa Rica. Such trees are regularly used in reforestation programmes at the local level, using soil preparations from these regions without any pre-inoculation. The nodules were surface-sterilized using sequential treatments with 70% ethanol and 2% sodium hypochlorite for 5 min, followed by rinsing with sterile distilled water. After maceration in sterile PBS (pH 7.4), bacterial suspensions were serially diluted (10^−1^ and 10^−2^) in peptone-yeast (PY) extract broth (5 g l^−1^ peptone, 1 g l^−1^ anhydrous dextrose, 0.5 g l^−1^ dipotassium phosphate, 0.2 g l^−1^ magnesium sulphate, 0.1 g l^−1^ sodium chloride), plated on PY-agar with cycloheximide to inhibit fungal growth and incubated at 28 °C for up to 3 weeks. Bacterial colonies were purified through streaking and cryopreserved in glycerol stocks at −80 °C.

DNA extraction from the four bacterial isolates was performed using the DNEasy PowerSoil Kit (Qiagen, USA), following manufacturer’s instructions. Sequencing was conducted with a NovaSeq PE150 platform (Illumina) at Novogene Co. (CA, USA) for all strains. Additionally, the genome of isolate 163b was sequenced using the PacBio RS II platform (Pacific Biosciences). Quality assessment and filtering of raw sequencing data were performed with fastp v0.20.1 (quality score >25) [6]. High-quality Illumina reads were assembled using SPAdes v3.15.4 [7]. For isolate 163b, a hybrid assembly was also performed using the same tool. Both assemblies were generated with the following k-mer values: 33, 55, 77, 99 and 127. Contigs shorter than 1,000 bp were filtered out using Seqtk v1.2 [8], assembly statistics were generated with QUAST v5.2.1 [9], completeness and redundancy of the assembled genomes were evaluated with CheckM2 v1.0.1 [10], and functional annotation of the genomes was performed using eggNOG-mapper v2 [11] (Table 1).

**Table 1.** Genome characteristics of the four *Bradyrhizobium* spp. isolates analysed in this work

Characteristic(s)	Isolates			
	*Bradyrhizobium* sp.LEGMi-117d	*Bradyrhizobium* sp.LEGMi-161a	*Bradyrhizobium* sp.LEGMi-163b	*Bradyrhizobium* sp.LEGMi-207a
**Host species**	*Pentaclethra macroloba*	*Chamaecrista nictitans*	*Erythrina fusca*	*Zygia engelsingii*
**Completeness (%**)	100	100	100	100
**Coverage (x**)	127	161	143	158.5
**No. Contigs**	93	55	60	75
**N50 (pb**)	359,180	492,614	600,185	417,354
**Genome length (bp**)	9,865,395	8,621,444	9,007,925	9,226,589
**CDS**	9216	8079	8341	8466
**GC content (%**)	62.79	63.63	63.81	63.13
***nod* genes**	*nodS*	*nodA, noB, nodS, nodIJ*	*nodA, nodB, nodIJ, nodS*	*nodA, nodB, nodC, nodD1, nodIJ, nodS, nodZ*
***nif* genes**	*nifA, nifB, nifD_1, nifD_2, nifK_1, nifK_2, nifS_1, nifS_2, nifW2, calB*	*nifA, nifB, nifD, nifH_1, nifH_2, nifK_1, nifK_2, nifS, nifW*	*nifA, nifB, nifD, nifH, nifK_1, nifK_2, nifS_1, nifS_2, nifW, calB*	*nifA, nifB, nifD, nifH_1, nifH_2, nifK_1, nifK_2, nifS_1, nifS_2, nifW2, calB*

We manually compiled a list of *Bradyrhizobium* genome accessions (primarily type strains of validly published species, selected based on the List of Prokaryotic names with Standing in Nomenclature taxonomy), which were then retrieved from GenBank using the GenFlow [12] pipeline. GenFlow automates genome downloading via NCBI command-line tools. All genomes were renamed for clarity with EDirect tool and processed with Anvi’o v7.1 [13]. To construct the 16S rRNA gene tree, we extracted 16S rRNA sequences from genomes using the anvi-get-sequences-for-hmm-hits function in Anvi’o v7.1 [13], which employs HMMER v3.3.2 [14] to identify genes. For the following strains: *Bradyrhizobium americanum* CMVU44^T^ (KU991833.1), * B. ferriligni* CCBAU 51502^T^ (KX683400.1), *B. ganzhouense* RITF806^T^ (JQ796661.2), *B. ingae* BR 10250^T^ (KF927043.1), * B. kavangense* 14-3^T^ (KP899562.1), *B. namibiense* 5-10^T^ (KX661401.2), *B. ripae* WR4^T^ (MF593081.1) and *B. subterraneum* 58 2-1^T^ (KP308152.1), genome accession numbers were not available in the NCBI database. Consequently, we retrieved their 16S rRNA gene sequences and aligned them with the 16S sequences extracted from the available *Bradyrhizobium* genomes. Sequence alignment was performed with MAFFT v7.397 [15], and a maximum likelihood phylogeny was inferred with IQ-TREE v2.2.0 [16], using the GTR+G+I model and 1,000 bootstrap replicates.

For phylogenomic analyses, coding sequences were annotated using Prodigal v2.6.3 [17], and Single Copy Core Genes across genomes were identified and concatenated with Anvi’o v7.1 [13], applying a geometric and functional index threshold of 0.8, to ensure the inclusion of gene clusters that are broadly conserved and functionally consistent. The geometric homogeneity index quantifies the uniformity of gene distribution across genomes, whereas the functional homogeneity index evaluates the consistency of gene function within clusters. Only gene clusters that met these homogeneity criteria were selected for downstream analyses. These sequences were then aligned using MAFFT v7.397 [15] and generated robust phylogenomic trees using FastTree v2.1.11 [18] with the JTT+CAT model and 1,000 bootstrap resampling. For both trees, visualization was performed using Interactive Tree of Life (iTOL) v6 [19].

To further evaluate the genomic relationships of the studied isolates in comparison to their closest relatives, the average nucleotide identity (ANI) value was calculated using the OrthoANIu [20] algorithm implemented in EzBioCloud [21], and digital DNA–DNA hybridization (dDDH) was determined using the Genome-to-Genome Distance Calculator 3.0 algorithm, based on formula 2 (*d4*), which calculates the proportion of identities over high-scoring segment pairs [22]. In Table 2, we show the most closely related species to each isolate based on ANI values, whereas Table 3 lists the corresponding dDDH values.

**Table 2.** ANI values between *Bradyrhizobium* strains under study and their closest validly published species

	Bradyrhizoboum hipponense aSej3 ^T^ GCF_008123965.1	Bradyrhizobium yuanmingense CCBAU 10071^T^ GCF 900094575 1	Bradyrhizobium centrolobii BR 10245 T ^T^ GCF_001641635.1	Bradyrhizobium barranii subsp. barranii 144 S4^T^ GCA_017565645.3
Bradyrhizobium *sp*. LEGMi-161a	86.05	92.11	85.85	87.15
Bradyrhizobium *sp*. LEGMi-207a	83.82	83.51	84.32	83.85
Bradyrhizobium *sp*. LEGMi-163b	86.86	87.18	86.44	90.13
Bradyrhizobium *sp*. LEGMi-117d	88.26	86.24	87.23	87.78

**Table 3.** dDDH (*d4*) values (in %) between *Bradyrhizobium* strains under study and their closest validly published species

	*B. hipponense aSej3*^T^GCF_008123965.1	*B*. yuanmingense CCBAU 10071^T^GCF 900094575 1	*B. centrolobii* BR 10245 T ^T^GCF_001641635.1	*B. barranii* subsp. barranii144S4^T^GCA_017565645.3
*Bradyrhizobium* sp. LEGMi-161a	31.40	48.40	31.30	35.30
*Bradyrhizobium* sp. LEGMi-207a	31.30	27.50	28.80	28.30
*Bradyrhizobium* sp. LEGMi-163b	33.00	33.80	39.50	41.30
*Bradyrhizobium* sp. LEGMi-117d	36.50	32.00	34.00	35.50

**Fig. 1.**
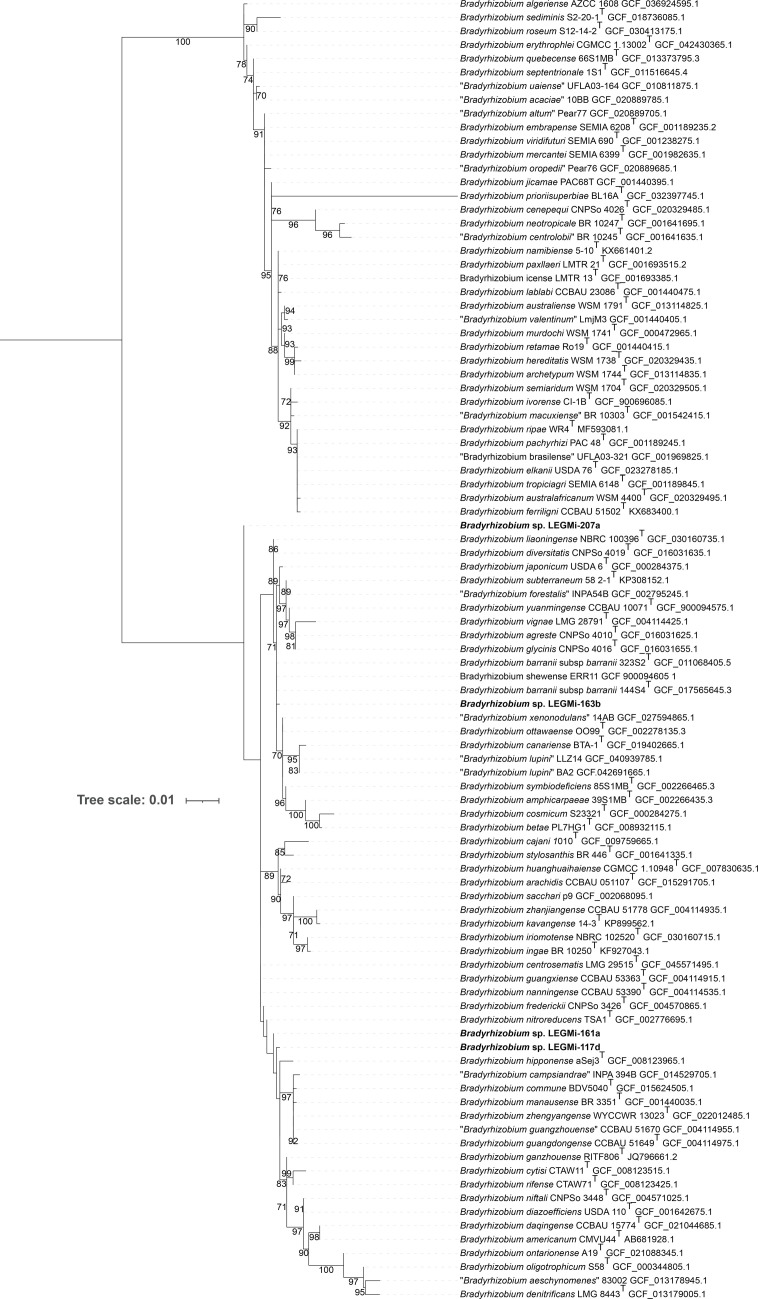
Maximum-likelihood 16S rRNA gene phylogenetic tree of *Bradyrhizobium* strains including four putative novel species. The phylogeny was generated with IQ-TREE v2 under the GTR+G+I nucleotide substitution model with 1,000 bootstrap replicates. The tree was visualized using iTOL v6. Reference genomes included in the tree were manually selected from the GenBank database. Accession numbers are provided next to each strain; for strains without a genome accession, the 16S rRNA gene accession number is shown instead. Bootstrap values ≥70% are shown at the nodes. The four putative new *Bradyrhizobium* species are highlighted in bold.

The four *Bradyrhizobium* isolates from this work are positioned in distinct clades relative to other *Bradyrhizobium* species in the phylogenetic trees, each supported by high confidence bootstrap values (Figs1  2). In addition, the isolates exhibited ANI and dDDH values below the commonly accepted species boundaries of 95–96% for ANI and 70% for dDDH when compared with their closest described *Bradyrhizobium* type strains (Tables2  3), supporting their classification as putative new species. Furthermore, the presence of diverse *nod* and *nif* genes suggests that these isolates have various capacities for nodulation and nitrogen fixation, possibly influenced by their host plants and environmental conditions. By characterizing isolates from underexplored tropical regions and host species, our study contributes to a broader understanding of the genomic diversity and evolutionary adaptations of *Bradyrhizobium*. Future phenotypic analyses will be crucial to confirm their species status and elucidate their ecological and biological roles.

**Fig. 2.**
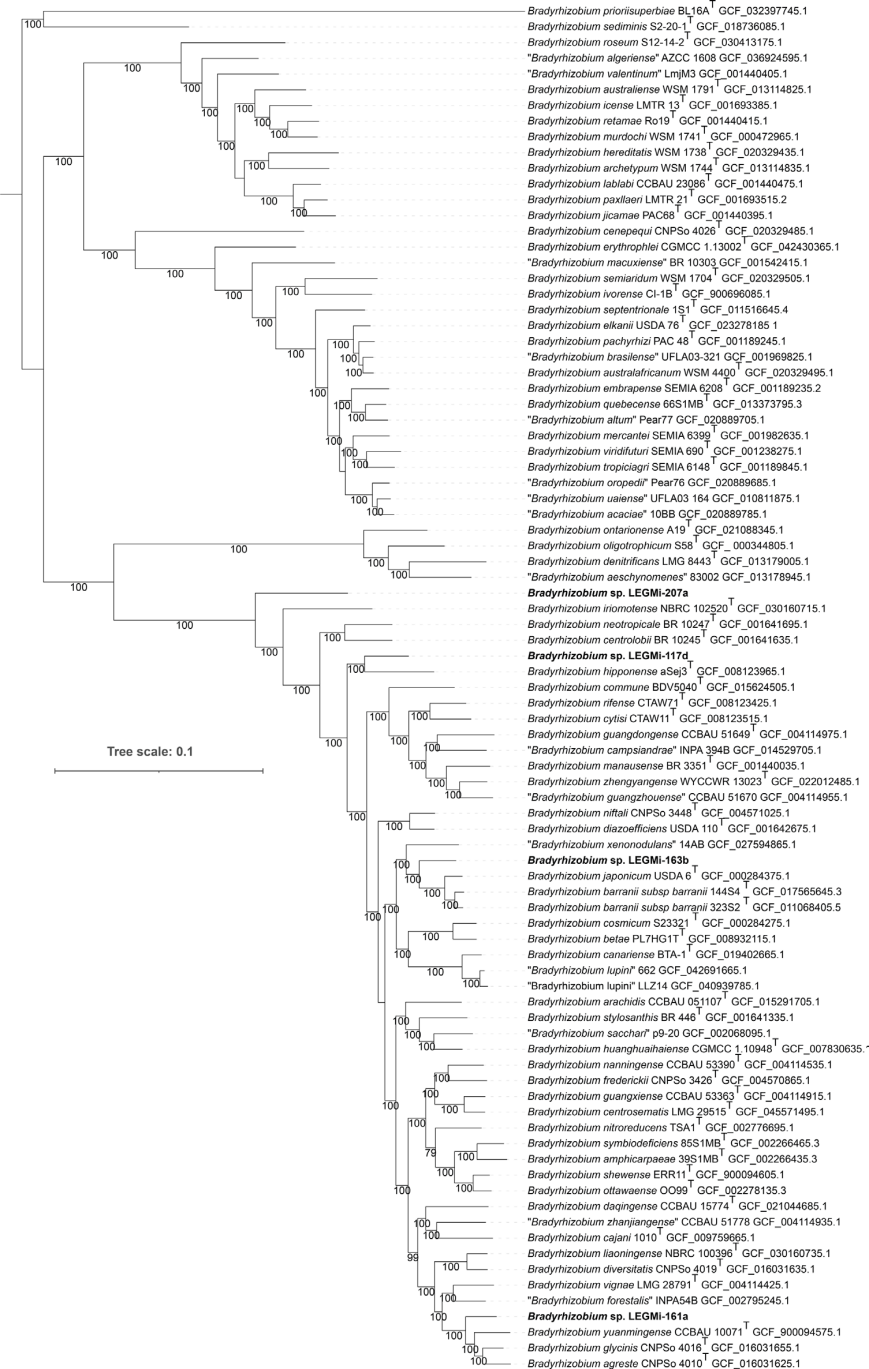
Phylogenomic tree of *Bradyrhizobium* spp. highlighting four potential new species obtained in this study. The phylogeny was constructed using FastTree [18] with the JTT+CAT model and 1,000 bootstrap replicates for robustness. A total of 745 single-copy orthologous gene sequences were used to build the concatenated alignment for tree inference. Visualization was performed using iTOL v6 [19], and the tree was rooted at midpoint. Reference genomes included in the tree were manually selected from the GenBank database, prioritizing type strains closely related to the query genomes. Accession numbers for each genome are indicated in labels, and bootstrap values superior to 70% are displayed at the nodes. The four putative new *Bradyrhizobium* species are highlighted in bold.

## Abbreviations

ANI: average nucleotide identity
dDDH: digital DNA–DNA hybridization
iTOL: interactive tree of life
PY: peptone yeast
